# Supplementary figures and images for: Genetic diversity of the Hungarian Gidran horse in two mitochondrial DNA markers
Source: PeerJ. 2016 May 2;4:e1894. doi: 10.7717/peerj.1894 (PMC4860319; doi:10.7717/peerj.1894)

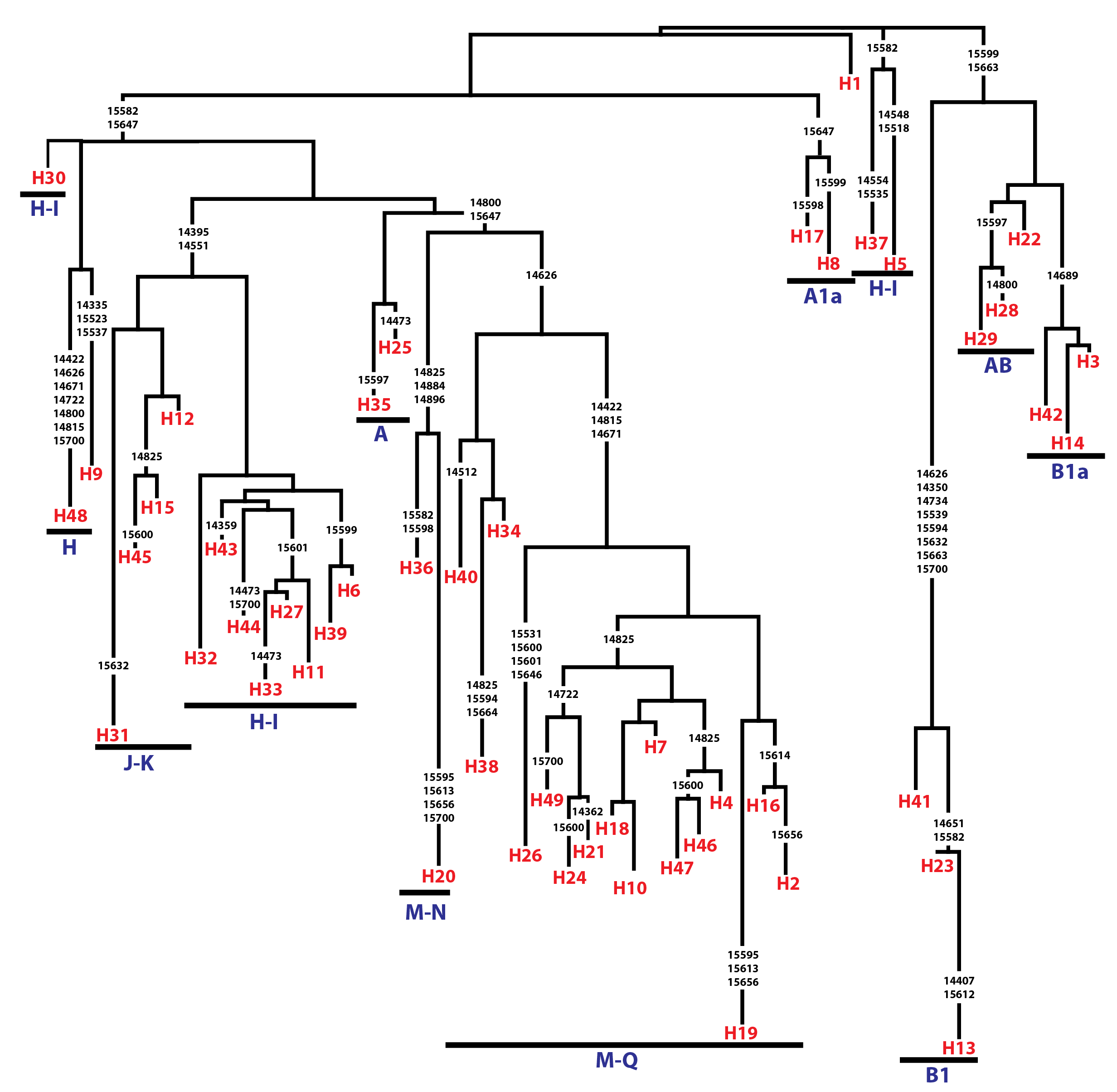

Supplement: Figure S1 — The polymorphic sites considered relative to the JN398377 reference sequence, which is identical with Haplotype 1 (H1). [file peerj-04-1894-s001.png]
