# Supplementary material for: Genetic diversity of the Hungarian Gidran horse in two mitochondrial DNA markers
Source: PeerJ. 2016 May 2;4:e1894. doi: 10.7717/peerj.1894 (PMC4860319; doi:10.7717/peerj.1894)
Supplement: Table S1 [file peerj-04-1894-s002.pdf]

		Polymorphic sites																							Individuals
Reference sequences	(GenBank accession nr.: X79547)	14335	14352	14361	14364	14397	14409	14424	14475	14514	14550	14553	14556	14628	14653	14673	14691	14724	14736	14802	14817	14827	14886	14896	
	(GenBank accession nr.: JN398377)	14333	14350	14359	14362	14395	14407	14422	14473	14512	14548	14551	14554	14626	14651	14671	14689	14722	14734	14800	14815	14825	14884	14894	
**Haplotypes**	**Number of mares**	C	C	C	G	C	C	T	T	C	C	A	T	G	A	G	C	T	C	T	A	G	C	G	
Ht1$_{CYTB}$	54	.	.	.	.	.	.	.	.	.	.	.	.	.	.	.	.	.	.	.	.	.	.	.	164G 165G 167G 171G 173G 175G 185G 190G 197G 198G 201G 108G 109G 110G 115G 117G 119G 126G 127G 13G 151G 153G 154G 157G 158G 163G 192G 196G 209G 235G 236G 238G 23G 244G 249G 250G 251G 252G 260G 3G 4G G15 G28 G31 G43 G48 G53 G54
Ht2$_{CYTB}$	49	.	.	.	.	.	.	C	.	.	.	.	.	A	.	A	.	.	.	C	G	.	.	.	166G 179G 180G 181G 182G 183G 184G 203G 103G 104G 105G 106G 112G 113G 123G 139G 140G 141G 142G 144G 145G 146G 147G 18G 199G 217G 218G 219G 21G 227G 228G 22G 231G 233G 241G 25G 38G G16 G44 G49 G51 G64 G68 G69 G75 G76 G80 G86 G89
Ht3$_{CYTB}$	9	.	.	.	.	.	.	.	.	.	.	.	.	.	.	.	T	.	.	.	.	.	.	.	168G 205G 206G 207G 208G 237G 259G 36G G81
Ht4$_{CYTB}$	12	.	.	.	.	.	.	C	.	.	.	.	.	A	.	A	.	.	.	C	G	A	.	.	169G 170G 174G 20G 240G 242G 248G G59 G63 G67 G78 G87
Ht5$_{CYTB}$	3	.	.	.	.	.	.	.	.	.	T	.	.	.	.	.	.	.	.	.	.	.	.	.	176G 155G G83
Ht6$_{CYTB}$	44	.	.	.	.	.	.	.	.	.	.	.	.	.	.	.	.	.	.	C	.	.	.	.	177G 189G 194G 200G 210G 211G 124G 125G 12G 136G 137G 138G 152G 178G 17G 220G 221G 222G 223G 224G 225G 226G 229G 230G 232G 234G 239G 253G 254G 255G 256G 257G 258G 2G 32G 39G 47G 7G G101 G27 G45 G46 G50 G85 G88
Ht7$_{CYTB}$	12	T	.	.	.	.	.	.	.	.	.	.	.	.	.	.	.	.	.	.	.	.	.	.	186G 191G 11G 150G 1G 41G G29 G57 G65 G66 G70 G8
Ht8$_{CYTB}$	5	.	.	.	A	.	.	C	.	.	.	.	.	A	.	A	.	.	.	C	G	.	.	.	187G 111G 160G 161G 162G
Ht9$_{CYTB}$	14	.	.	.	.	.	.	.	.	.	.	.	.	.	.	.	.	.	.	C	.	A	.	.	188G 204G 131G 132G 133G 134G 148G 149G 40G G19 G55 G58 G6 G79
Ht10$_{CYTB}$	5	.	.	.	.	T	.	.	.	.	.	G	.	.	.	.	.	.	.	C	.	.	.	.	193G 195G 128G 129G G71
Ht11$_{CYTB}$	1	.	T	.	.	.	A	.	.	.	.	.	.	A	G	.	.	.	T	.	.	.	.	.	202G
Ht12$_{CYTB}$	11	.	.	.	.	T	.	.	.	.	.	G	.	.	.	.	.	.	.	C	.	A	.	.	100G 101G 102G 92G 93G 94G 95G 96G 97G 98G 99G
Ht13$_{CYTB}$	7	.	.	.	.	.	.	.	.	.	.	.	.	.	.	.	.	.	.	C	.	A	T	A	114G 143G 245G 33G 34G 35G G77
Ht14$_{CYTB}$	5	.	.	.	.	.	.	C	.	.	.	.	.	A	.	A	.	C	.	C	G	.	.	.	116G 120G 121G G52 G72
Ht15$_{CYTB}$	2	.	T	.	.	.	.	.	.	.	.	.	.	A	G	.	.	.	T	.	.	.	.	.	118G 37G
Ht16$_{CYTB}$	3	.	.	.	.	.	.	.	C	.	.	.	.	.	.	.	.	.	.	C	.	.	.	.	122G 130G 91G
Ht17$_{CYTB}$	1	.	.	.	.	.	.	.	.	.	.	.	.	A	.	.	.	.	.	C	.	.	.	.	135G
Ht18$_{CYTB}$	1	.	.	.	.	.	.	.	.	.	.	.	C	.	.	.	.	.	.	.	.	.	.	.	156G
Ht19$_{CYTB}$	3	.	.	.	.	.	.	.	.	.	.	.	.	A	.	.	.	.	.	C	.	A	.	.	159G G73 G82
Ht20$_{CYTB}$	1	.	.	.	.	.	.	.	.	.	.	.	.	.	.	.	.	.	.	C	.	C	.	.	216G
Ht21$_{CYTB}$	1	.	.	.	.	.	.	.	.	T	.	.	.	A	.	.	.	.	.	C	.	.	.	.	243G
Ht22$_{CYTB}$	1	.	T	.	.	.	.	.	.	.	.	.	.	A	.	.	.	.	T	.	.	.	.	.	247G
Ht23$_{CYTB}$	5	.	.	.	.	.	.	.	.	.	.	.	.	.	.	.	T	.	.	.	.	A	.	.	24G G14 G26 G30 G84
Ht24$_{CYTB}$	1	.	.	T	.	.	.	.	.	.	.	.	.	.	.	.	.	.	.	C	.	.	.	.	90G

Table S1: Variable site positions in 686 bp long mtDNA *CYTB* sequence of 24 Gidran haplotypes. New haplotypes are emphasized by gray colour.
