# Supplementary material for: Genetic diversity of the Hungarian Gidran horse in two mitochondrial DNA markers
Source: PeerJ. 2016 May 2;4:e1894. doi: 10.7717/peerj.1894 (PMC4860319; doi:10.7717/peerj.1894)
Supplement: Table S2 [file peerj-04-1894-s003.pdf]

		Polymorphic sites																									
Reference sequences	(GenBank accession nr.: X79547)	15521	15526	15534	15538	15540	15542	15544	15585	15597	15598	15600	15601	15602	15603	15604	15615	15616	15617	15635	15649	15650	15659	15666	15667	15686	15703	Individuals
	(GenBank accession nr.: JN398377)	15518	15523	15531	15535	15537	15539	15541	15582	15594	15595	15597	15598	15599	15600	15601	15612	15613	15614	15632	15646	15647	15656	15663	15664	15683	15700	
Haplotypes	Number of mares	G	T	C	A	A	C	T	G	A	T	G	T	C	T	G	A	A	T	C	A	A	T	G	A	A	T	
Ht1$_{D-loop}$	12	.	.	.	.	.	.	.	.	.	.	.	.	.	.	.	.	.	.	.	.	.	.	.	.	.	.	115G 153G 154G 15G 185G 190G 192G 196G 197G 198G 201G 23G 249G 250G 252G 28G 31G 3G 43G 48G 53G 5G 60G 61G 62G
Ht2$_{D-loop}$	4	.	.	.	.	.	.	.	.	.	.	.	.	T	.	.	.	.	C	.	.	.	.	.	.	.	.	103G 104G 105G 106G
Ht3$_{D-loop}$	15	.	.	.	.	.	.	.	.	.	.	.	.	T	.	.	.	.	.	.	.	G	.	.	.	.	.	108G 109G 164G 165G 167G 171G 173G 175G 209G 235G 238G 260G 4G 56G 9G
Ht4$_{D-loop}$	22	.	.	T	.	.	.	.	A	.	.	.	.	T	C	A	.	.	.	.	G	.	.	.	.	.	.	10G 123G 124G 130G 131G 132G 133G 134G 136G 137G 148G 149G 188G 19G 204G 2G 55G 58G 6G 79G 7G 85G
Ht5$_{D-loop}$	5	.	.	.	.	.	.	.	.	.	.	.	C	T	.	.	.	.	.	.	.	.	.	.	.	.	.	110G 160G 161G 162G 187G
Ht6$_{D-loop}$	47	.	.	.	.	.	.	.	A	.	.	.	C	T	.	.	.	.	.	.	.	.	.	.	.	.	.	111G 112G 116G 120G 122G 139G 140G 141G 143G 144G 145G 146G 147G 169G 170G 172G 174G 179G 180G 181G 182G 183G 184G 18G 199G 203G 20G 217G 218G 219G 227G 228G 231G 233G 240G 241G 242G 248G 25G 38G 52G 67G 76G 78G 86G 87G 89G
Ht7$_{D-loop}$	7	.	.	.	.	.	.	.	.	.	C	.	.	T	.	.	.	G	.	.	.	.	C	.	.	.	C	113G 114G 142G 245G 33G 34G 77G
Ht8$_{D-loop}$	25	.	.	.	.	.	.	.	A	.	.	.	.	T	.	.	.	.	.	.	.	.	.	.	.	.	.	100G 101G 102G 127G 193G 195G 71G 92G 93G 97G 98G 99G
Ht9$_{D-loop}$	8	.	.	.	.	.	.	.	A	.	.	.	.	.	.	.	.	.	.	.	.	G	.	A	.	.	.	117G 119G 13G 151G 157G 158G 54G 74G
Ht10$_{D-loop}$	2	.	.	.	.	.	T	.	A	G	.	.	.	T	.	.	.	.	.	T	.	G	.	A	.	.	C	118G 37G
Ht11$_{D-loop}$	11	.	C	.	.	G	.	.	A	.	.	.	.	T	.	.	.	.	.	.	G	.	.	.	.	.	.	11G 150G 186G 191G 1G 29G 41G 57G 66G 70G 8G
Ht12$_{D-loop}$	2	.	.	.	.	.	.	.	A	.	.	.	C	T	C	.	.	.	.	.	.	.	.	.	.	.	.	121G 59G
Ht13$_{D-loop}$	4	.	.	.	.	.	.	.	A	.	.	A	.	.	.	.	.	.	.	.	.	G	.	A	.	.	.	125G 126G 163G 236G
Ht14$_{D-loop}$	1	.	.	.	.	.	.	C	.	.	.	.	.	T	.	.	.	.	.	T	.	G	.	.	.	G	.	128G
Ht15$_{D-loop}$	2	.	.	T	.	.	.	.	A	.	.	.	.	T	C	.	.	.	.	.	G	.	.	.	.	.	.	129G 90G
Ht16$_{D-loop}$	35	.	.	T	.	.	.	.	A	.	.	.	.	.	C	.	.	.	.	.	G	.	.	.	.	.	.	12G 152G 177G 178G 17G 189G 194G 200G 210G 211G 216G 220G 221G 222G 223G 224G 225G 226G 229G 230G 232G 234G 239G 253G 254G 255G 256G 257G 258G 27G 32G 45G 46G 50G 88G
Ht17$_{D-loop}$	1	.	.	.	.	.	.	.	.	.	.	.	.	T	.	A	.	.	.	.	.	.	.	.	.	.	C	135G
Ht18$_{D-loop}$	1	.	.	.	.	.	.	.	A	.	.	A	C	T	.	.	.	.	.	.	.	.	.	.	.	.	.	138G
Ht19$_{D-loop}$	7	.	.	.	.	.	.	.	.	.	.	.	.	.	.	.	.	.	.	.	.	G	.	A	.	.	.	14G 168G 24G 259G 26G 30G 84G
Ht20$_{D-loop}$	3	A	.	.	.	.	.	.	A	.	.	.	.	T	.	.	.	.	.	.	.	G	.	.	.	.	.	155G 176G 83G
Ht21$_{D-loop}$	1	.	.	.	G	.	.	.	A	.	.	.	.	T	.	.	.	.	.	.	.	G	.	.	.	.	.	156G
Ht22$_{D-loop}$	3	.	.	.	.	.	.	.	.	G	.	.	.	T	.	A	.	.	.	.	.	.	.	.	G	.	C	159G 73G 82G
Ht23$_{D-loop}$	10	.	.	.	.	.	.	.	.	.	.	.	.	T	.	.	.	.	C	.	.	.	C	.	.	.	.	166G 16G 21G 44G 51G 64G 68G 69G 75G 80G
Ht24$_{D-loop}$	1	.	.	.	.	.	T	.	A	G	.	.	.	T	.	.	G	.	.	T	.	G	.	A	.	.	C	202G
Ht25$_{D-loop}$	7	.	.	.	.	.	.	.	.	.	.	A	.	.	.	.	.	.	.	.	.	G	.	A	.	.	.	205G 206G 207G 208G 237G 36G 81G
Ht26$_{D-loop}$	2	.	.	.	.	.	.	.	A	.	.	.	.	T	.	A	.	.	.	.	.	.	.	.	.	.	C	243G
Ht27$_{D-loop}$	1	.	.	.	.	.	T	.	.	G	.	.	.	T	.	.	.	.	.	T	.	G	.	A	.	.	C	246G 247G
Ht28$_{D-loop}$	1	.	.	.	.	.	.	.	A	.	.	.	C	T	A	.	.	.	.	.	.	.	.	.	.	.	.	63G
Ht29$_{D-loop}$	1	.	.	.	.	.	.	.	.	.	.	.	.	T	.	.	.	.	C	.	G	.	C	.	.	.	.	65G
Ht30$_{D-loop}$	1	.	.	.	.	.	.	.	A	.	.	.	.	T	.	.	.	.	.	.	.	.	.	.	.	.	C	72G
Ht31$_{D-loop}$	1	.	.	T	.	.	.	.	A	.	.	.	.	T	C	.	.	.	.	.	G	.	.	.	.	.	C	91G
Ht32$_{D-loop}$	3	.	.	.	.	.	.	.	A	.	.	.	.	T	C	.	.	.	.	.	.	.	.	.	.	.	.	94G 95G 96G

Table S2: Variable site positions in 202 bp of mtDNA D-loop region of 32 Gidran haplotypes. New haplotypes are emphasized by gray colour.
