# Supplementary material for: Genetic diversity of the Hungarian Gidran horse in two mitochondrial DNA markers
Source: PeerJ. 2016 May 2;4:e1894. doi: 10.7717/peerj.1894 (PMC4860319; doi:10.7717/peerj.1894)
Supplement: Table S3 [file peerj-04-1894-s004.pdf]

CYTB haplotyes	GenBank accession numbers	Qi, A. Et al., 2012* (PopSet: 426260659)	Ning, T. et al., 2009[a] (PopSet: 262348272)	Ning, T. et al., 2008[a] (PopSet: 183181282)	Achilli, A., et al., 2013[b]** (PopSet: 374725290)	Peng et al., 2015[c] (DomeTree)
**Ht1CYTB**	KT792934	A1, A2, B1	101, 100, 98, 95, 94, 76, 70, 67, 59	hap30	A, B, C, D	H
**Ht2CYTB**	KT792935	M1, M2, M4, M5, M6, N2, N3, N4	80, 79, 57	hap13, hap12	N, M	M-N
**Ht3CYTB**	KT792936	-	93, 65	-	B	B1a2
**Ht4CYTB**	KT792937	-	-	-	-	
**Ht5CYTB**	KT792938	-	-	-	-	H
**Ht6CYTB**	KT792939	L9, L8, L6, L5, L4, L36, L35, L32, L31, L30, L3, L27, L23, L21, L19, L18, L17, L16, L15, L14, L13, L12, L10, L1	89	hap17	L	A1b
**Ht7CYTB**	KT792940	H1	-	hap43, hap44	H	H
**Ht8CYTB**	KT792941	-	-	-	-	M-N
**Ht9CYTB**	KT792942	L29	-	-	-	A-Q
**Ht10CYTB**	KT792943	-	48, 50, 97	-	K, J	JK
**Ht11CYTB**	KT792944	-	-	-	-	G1a
**Ht12CYTB**	KT792945	-	-	-	J	J2
**Ht13CYTB**	KT792946	-	92	hap37, hap38	R	A-Q
**Ht14CYTB**	KT792947	-	-	-	-	M-N
**Ht15CYTB**	KT792948	-	-	-	G	G1a
**Ht16CYTB**	KT792949	-	-	-	-	G1b
**Ht17CYTB**	KT792950	-	58	hap24, hap35, hap36	Q,P	M-Q
**Ht18CYTB**	KT792951	I9, I7, I6, I5, I4, I3, I16, I15, I14, I13, I12, I11, I10	53	-	I	H
**Ht19CYTB**	KT792952	-	-	hap31	O	M-Q
**Ht20CYTB**	KT792953	-	-	-	-	J2
**Ht21CYTB**	KT792954	-	-	-	-	M-Q
**Ht22CYTB**	KT792955	G8, G7, G6, G5, G4, G3, G2, G1	-	hap24, hap35, hap36	G, F	G3
**Ht23CYTB**	KT792956	-	-	-	-	D
**Ht24CYTB**	KT792957	-	-	hap18	L	L1b

Table S3: The nominations of the detected *CYTB* haplotypes in different horse studies. (*Unpublished data; **Haplotypes were clustered into haplogroups)

References:

[a]Ning T, Li J, Lin K, Xiao H, Wylie S, Hua S, Li H, and Zhang Y-P. 2014. Complex Evolutionary Patterns Revealed by Mitochondrial Genomes of the Domestic Horse. Current molecular medicine 14:1286-1298.

[b]Achilli A, Olivieri A, Soares P, Lancioni H, Kashani BH, Perego UA, Nergadze SG, Carossa V, Santagostino M, and Capomaccio S. 2012. Mitochondrial genomes from modern horses reveal the major haplogroups that underwent domestication. Proceedings of the National Academy of Sciences 109:2449-2454.

[c]Peng MS, Fan L, Shi NN, Ning T, Yao YG, Murphy RW, Wang WZ, and Zhang YP. 2015. DomeTree: a canonical toolkit for mitochondrial DNA analyses in domesticated animals. Molecular ecology resources.
