# Supplementary material for: Genetic diversity of the Hungarian Gidran horse in two mitochondrial DNA markers
Source: PeerJ. 2016 May 2;4:e1894. doi: 10.7717/peerj.1894 (PMC4860319; doi:10.7717/peerj.1894)
Supplement: Table S4 [file peerj-04-1894-s005.pdf]

D-loop haplotypes	GenBank accession numbers	Ning, T. et al. 2011* (PopSet: 401761235)	Qi, A. et al. 2012* (PopSet: 42626059)	Flannery, A.R. et al., 2003* (PopSet: 29650781)	Moridi, M. et al., 2013[a] (PopSet: 356650048)	Juras, R. et al., 2004* (PopSet: 50916302)	Achilli, A. et al., 2013[b]** (PopSet: 374725290)	Peng M.S. et al., 2015[c] (DomeTree)
**Ht1$_{D\text{-loop}}$**	KT818898	Hap_322, Hap_307, Hap_306, Hap_287, Hap_276, Hap_250	A2, A1	nk3, hf1, cs8, be2	-	-	-	AB
**Ht2$_{D\text{-loop}}$**	KT818892	Hap_266	M1	-	-	PI1	M	AB
**Ht3$_{D\text{-loop}}$**	KT818893	Hap_290, Hap_242	-	fr5, fr6	-	HU1	C	J1
**Ht4$_{D\text{-loop}}$**	KT818894	Hap_238, Hap_227, Hap_167	L8, L4, L37, L34, L33, L28, L13, L11, L27, L26, L24, L23, L22, L20, L2, L17, L16, L10, L1	fr1, d5, d4, be8, be7	-	PH3	-	J1
**Ht5$_{D\text{-loop}}$**	KT818895	-	N4, N3, N2, N1	ex6, cj2	-	-	N	L
**Ht6$_{D\text{-loop}}$**	KT818896	-	N4, N3, N2, N1	-	-	PO2, GT1	-	L
**Ht7$_{D\text{-loop}}$**	KT818897	-	-	-	-	-	-	R
**Ht8$_{D\text{-loop}}$**	KT818891	Hap_272, Hap_230, Hap_216, Hap_189, Hap_163	N4, N3, N2, N1	-	H40	-	-	A1d
**Ht9$_{D\text{-loop}}$**	KT818899	Hap_215	B2	ar2	-	HA2	B	B1a
**Ht10$_{D\text{-loop}}$**	KT818900	Hap_213, Hap_188, Hap_176	-	-	-	-	G	G1
**Ht11$_{D\text{-loop}}$**	KT818901	-	H1	-	-	PV3	H	H-I
**Ht12$_{D\text{-loop}}$**	KT818902	-	-	-	-	-	-	L
**Ht13$_{D\text{-loop}}$**	KT818903	Hap_243	-	ar2	-	-	-	J2
**Ht14$_{D\text{-loop}}$**	KT818904	-	-	-	-	-	-	K
**Ht15$_{D\text{-loop}}$**	KT818905	-	L7	gr4	-	TK1	-	L
**Ht16$_{D\text{-loop}}$**	KT818906	-	L6, L5, L31, L30, L29, L18, L15, L12	tb4, tb3, tb2, hf2	H10	HU2	L	L
**Ht17$_{D\text{-loop}}$**	KT818907	Hap_291, Hap_225	-	sh1, sh4, gr5, at5, at6	-	-	-	OP
**Ht18$_{D\text{-loop}}$**	KT818908	-	-	cb3	H41	-	-	J2
**Ht19$_{D\text{-loop}}$**	KT818909	Hap_298, Hap_296, Hap_246, Hap_233	B1	ar6	H18	GT3	B	B1
**Ht20$_{D\text{-loop}}$**	KT818910	Hap_300, Hap_229	-	-	-	-	-	D
**Ht21$_{D\text{-loop}}$**	KT818911	-	I11, I12	-	H28	-	-	I
**Ht22$_{D\text{-loop}}$**	KT818912	-	-	cj8	-	PO1, FH1	P	OP
**Ht23$_{D\text{-loop}}$**	KT818913	Hap_341, Hap_283	M6, M5, M4, M3, M2	nk4, gr3, cb2, cb5, be5, at1	-	-	M	AB
**Ht24$_{D\text{-loop}}$**	KT818914	Hap_222	-	-	-	-	-	G1a
**Ht25$_{D\text{-loop}}$**	KT818915	-	-	-	-	-	-	J2
**Ht26$_{D\text{-loop}}$**	KT818916	Hap_ 338	-	-	H32, H21	-	Q	G
**Ht27$_{D\text{-loop}}$**	KT818917	Hap_310	-	so1, so7, ar5	-	HA3	G	G
**Ht28$_{D\text{-loop}}$**	KT818918	-	-	-	-	-	-	L
**Ht29$_{D\text{-loop}}$**	KT818919	-	-	-	-	-	-	AB
**Ht30$_{D\text{-loop}}$**	KT818920	Hap_272, Hap_230, Hap_216, Hap_189, Hap_163	-	nk6, at3	-	-	Q	A1d
**Ht31$_{D\text{-loop}}$**	KT818921	-	-	gr4	-	TH1	L	G
**Ht32$_{D\text{-loop}}$**	KT818922	-	-	-	-	-	-	A3

Table S4: The nominations of the detected D-loop haplotypes in different horse studies. (*Unpublished data; **Haplotypes were clustered into haplogroups)

References:

[a]Moridi M, Masoudi A, Vaez Torshizi R, and Hill E. 2013. Mitochondrial DNA D-loop sequence variation in maternal lineages of Iranian native horses. Animal Genetics 44: 209-213.

[b]Achilli A, Olivieri A, Soares P, Lancioni H, Kashani BH, Perego UA, Nergadze SG, Carossa V, Santagostino M, and Capomaccio S. 2012. Mitochondrial genomes from modern horses reveal the major haplogroups that underwent domestication. Proceedings of the National Academy of Sciences 109:2449-2454.

[c]Peng MS, Fan L, Shi NN, Ning T, Yao YG, Murphy RW, Wang WZ, and Zhang YP. 2015. DomeTree: a canonical toolkit for mitochondrial DNA analyses in domesticated animals. Molecular ecology resources.
