# Supplementary material for: Genetic diversity of the Hungarian Gidran horse in two mitochondrial DNA markers
Source: PeerJ. 2016 May 2;4:e1894. doi: 10.7717/peerj.1894 (PMC4860319; doi:10.7717/peerj.1894)
Supplement: Table S5 [file peerj-04-1894-s006.pdf]

Gidran mare families	Date of establishment	Founder mares
**mezőhegyesi 1**	1783	original Holsteiner mare (Nr. 99.)
**mezőhegyesi 2**	1783	original Holsteiner mare (Nr. 123.)
**mezőhegyesi 3**	1785	original Hungarian mare (Nr. 162.)
**mezőhegyesi 4**	1788	original Moldavian mare (Nr. 722.)
**mezőhegyesi 5**	1783	original Transylvanian mare (Nr. 265.)
**mezőhegyesi 6**	1805	original Moldavian mare (Nr. 743.)
**mezőhegyesi 7**	1807	original Moldavian mare (Nr. 596.)
**mezőhegyesi 8**	1814	original Hungarian mare (Nr. 553)
**mezőhegyesi 9**	1814	original Hungarian mare (Nr. 574)
**mezőhegyesi 11**	1815	original Hungarian mare (Nr. 819)
**mezőhegyesi 12**	1814	original Hungarian mare (Nr. 518)
**mezőhegyesi 13**	1805	original Moldvai mare (Nr. 405.)
**mezőhegyesi 14**	1786	original Transylvanian mare (Nr. 59.)
**mezőhegyesi 15**	1810	original Hungarian mare (Nr. 270)
**mezőhegyesi 17**	1810	74 Tifle (Arabian mare from Bábolna)
**mezőhegyesi 18**	1920	303 Szennyes (English half-bred mare)
**mezőhegyesi 19**	1928	Luza (English half-bred mare)
**mezőhegyesi 21**	1812	206 Carola
**borodi 1**	1956	52 Sütvény
**borodi 2**	1979	56 Regöly Kényeskanca
**borodi 3**	1979	58 Regöly Jólány
**borodi 5**	1964	5 Balotaszállás Gidran-720 Maca
**borodi 6**	1961	78 Sütvény Ozora (Mályva)
**borodi 7**	1967	82 Sütvény Gidran (Szimia)
**borodi 14**	1968	14 Regöly Gidran (Iram)
**borodi 17**	1960	17 Bátya Gidran
**borodi 18**	1972	18 Fürged Szellő
**borodi 19**	1962	19 Hajós Juci
**népies 9**	1852	120 Queen
**népies 22**	1991	1151 Gidran Dalova
**népies 23**	1991	Maxim Gidran-80 (Perzsi)

Table S5. Summary of the 31 mare families according to the Gidran studbook
